# Treatment of Cystic Fibrosis Patients Homozygous for *F508del* with Lumacaftor-Ivacaftor (Orkambi^®^) Restores Defective CFTR Channel Function in Circulating Mononuclear Cells

**DOI:** 10.3390/ijms21072398

**Published:** 2020-03-31

**Authors:** Maria Favia, Crescenzio Gallo, Lorenzo Guerra, Domenica De Venuto, Anna Diana, Angela Maria Polizzi, Pasqualina Montemurro, Maria Addolorata Mariggiò, Giuseppina Leonetti, Antonio Manca, Valeria Casavola, Massimo Conese

**Affiliations:** 1 Department of Biosciences, Biotechnologies and Biopharmaceutics, University of Bari, 70125 Bari, Italy; mariafavia@hotmail.com (M.F.); lorenzo.guerra1@uniba.it (L.G.); valeria.casavola@uniba.it (V.C.); 2 Department of Clinical and Experimental Medicine, University of Foggia, 71122 Foggia, Italy; crescenzio.gallo@unifg.it; 3 Cystic Fibrosis Regional Center, Department of Biomedical and Human Oncology, Pediatrics Section, U.O. “B. Trambusti”, Policlinico, University of Bari, 70124 Bari, Italy; domenica.devenuto@policlinico.ba.it (D.D.V.); susy.leonetti@virgilio.it (G.L.); amanca955@gmail.com (A.M.); 4 UOC Laboratorio di Genetica Medica, Department of Biomedical and Human Oncology, Policlinico, University of Bari, 70124 Bari, Italy; diana_anna@libero.it (A.D.); angelamaria.polizzi@uniba.it (A.M.P.); 5 Department of Biomedical Sciences and Human Oncology, Section of General Pathology, University of Bari, 70124 Bari, Italy; pasqualina.montemurro@uniba.it (P.M.); mariaaddolorata.mariggio@uniba.it (M.A.M.); 6 Department of Medical and Surgical Sciences, University of Foggia, 71122 Foggia, Italy

**Keywords:** cystic fibrosis, Orkambi^®^, CFTR, mononuclear cells, BMI, sweat chloride, FEV_1_%

## Abstract

The treatment of cystic fibrosis (CF) patients homozygous for the *F508del* mutation with Orkambi^®^, a combination of a corrector (lumacaftor) and a potentiator (ivacaftor) of the mutated CFTR protein, resulted in some amelioration of the respiratory function. However, a great variability in the clinical response was also observed. The aim of this study was to evaluate the response to Orkambi^®^ in a small cohort of F508del/F508del patients (*n* = 14) in terms of clinical and laboratory parameters, including ex vivo CFTR activity in mononuclear cells (MNCs), during a 12-month treatment. Patients responded with an increase in percent predicted forced expiratory volume in 1 s (FEV_1_%) and body mass index (BMI) as well as with a decrease in white blood cell (WBC) total counts and serum C-reactive protein (CRP) levels, although not significantly. Sweat chloride and CFTR-dependent chloride efflux were found to decrease and increase, respectively, as compared with pre-therapy values. CFTR and BMI showed a statistically significant correlation during Orkambi^®^ treatment. Clustering analysis showed that CFTR, BMI, sweat chloride, FEV_1_%, and WBC were strongly associated. These data support the notion that CFTR-dependent chloride efflux in MNCs should be investigated as a sensitive outcome measure of Orkambi^®^ treatment in CF patients.

## 1. Introduction

Cystic fibrosis (CF) is an autosomal recessive disorder due to mutations occurring in the *CFTR* (*CF Transmembrane Conductance Regulator*) gene, encoding for a chloride-conducting channel located at the apical membrane of epithelial cells. The lack/dysfunction of the CFTR protein leads to progressive fat and vitamin malabsorption and failure to thrive, abnormal salt concentration in sweat, and progressive lung failure due to infection by opportunistic pathogens and mucosal chronic inflammation [1].

CFTR is a glycosylated protein that functions as a chloride channel regulated by cAMP and protein kinase A (PKA)-mediated phosphorylation [2]. In normal cells, after passing the endoplasmic reticulum quality control, CFTR is translocated to the Golgi complex where it is fully glycosylated and subsequently transported and inserted into the apical membrane of polarized cells, where it is subjected to endocytosis and then recycled to the plasma membrane or targeted for lysosomal degradation [3,4,5]. The most frequent CFTR mutation leads to the deletion of phenylalanine at position 508 of CFTR (*F508del*), resulting in a folding defect and premature degradation by the ubiquitin-proteasome system [6,7,8]. Consequently, in the respiratory system of patients affected by CF, there is a defective Cl^−^ secretion, a NaCl hyperabsorption, a depletion of airway surface liquid, and a secondary failure of mucociliary clearance [9] associated with an exaggerated immune response and impairment of lung function [10].

The pharmacological corrector VX-809 (now lumacaftor) was found to interact directly with the MSD1 domain of CFTR and partially rescue the functional expression of F508del-CFTR to the cell surface in heterologous expression systems [11,12,13]. Together with the potentiator VX-770 (now ivacaftor), a drug that facilitates the opening of the F508del-CFTR channel present on the plasma membrane channel activity [14], lumacaftor has been found to enhance the functional activity of F508del-CFTR in pre-clinical studies conducted on primary bronchial cell cultures and rectal biopsy-derived organoids [11,15]. In 2015, the U.S. FDA approved ivacaftor-lumacaftor (Orkambi^®^) for the use in patients 12 years of age or older and homozygous for the *F508del* CFTR mutation [16]. Orkambi^®^ treatment has been demonstrated to be associated with an improvement in pulmonary function and reduction in respiratory exacerbations and its use was recently extended to include those 6–11 years old [17]. The monitoring of treatment in 1030 patients up to 96 weeks has revealed that Orkambi^®^ is safe and allows for a prolonged slower rate of FEV_1_ decline than in matched registry controls [18]. However, while Orkambi^®^ therapy influences different factors, such as the F508del- CFTR stability or the mucociliary clearance [19,20], it is associated with variable clinical responsiveness [21] suggesting that its efficacy may differ according to the patients’ conditions, including genetics. These findings highlight the importance of finding new biomarkers predictive of an individual patient’s response.

In the search of novel and non-invasive biomarkers, which should be predictive of the therapeutic response in CF patients, we recently demonstrated [22] that human peripheral blood monocytes (MNCs) isolated from CF patients treated with ivacaftor could represent an important tool for the analysis of CFTR-dependent chloride efflux rescue and to evaluate the therapeutic response in CF patients. Furthermore, the correlation between MNC chloride efflux with FVC and FEV_1_ indicates that this test could be a surrogate marker similar to the respiratory function parameters. In this study, we sought to demonstrate that the treatment combination ivacaftor-lumacaftor (Orkambi^®^) was able to correct the CFTR-dependent chloride efflux in CF people homozygous for *F508del*. Overall, the data presented in this study support the notion that CFTR-dependent chloride efflux in MNCs should be investigated as a sensitive outcome measure of Orkambi^®^ treatment in CF patients.

## 2. Results

### 2.1. Clinical Monitoring during Orkambi^®^ Treatment

We recruited fourteen CF patients homozygous for the F508del mutation and variable lung disease as judged from FEV_1_% predicted (FEV_1_%). At the baseline (pre-therapy), these patients were characterized by high sweat chloride and high CRP levels, and were underweight (Table 1). They were also characterized by systemic inflammatory response (i.e., high CRP).

**Figure 1:**
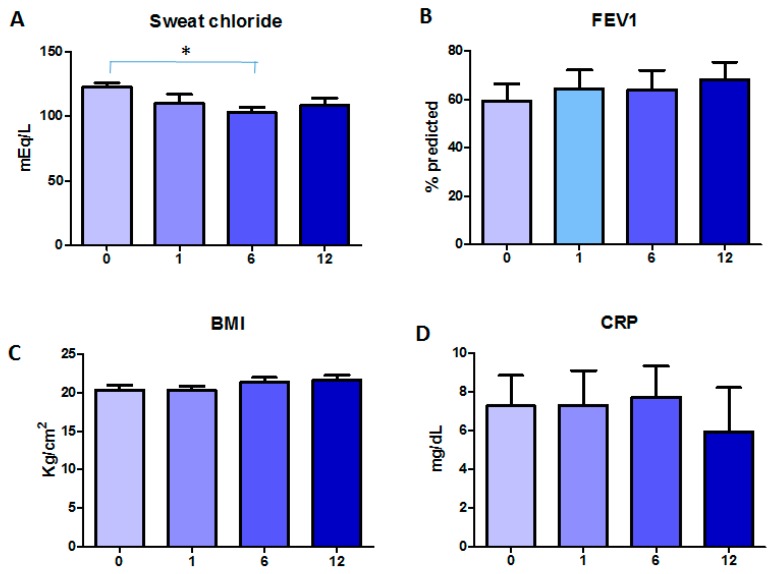
Changes in clinical parameters during Orkambi^®^ treatment: (**A**) sweat chloride levels; (**B**) FEV_1_%; (**C**) BMI; and (**D**) CRP. Data are shown as mean ± SEM.* *p* < 0.05.

Follow-up of these patients was at 1, 6, and 12 months of Orkambi*^®^* treatment. Figure 1 depicts the changes of clinical parameters in time. Compared with starting levels (122.9 ± 3.2 mEq/L (mean ± SEM)), sweat chloride showed a decreasing trend over time, with a significant difference at six months (*p* < 0.05), while BMI and FEV_1_% increased in time, although only by trend. In particular, BMI increased from 20.4 ± 0.6 to 21.7 ± 0.6 Kg/m^2^, and FEV_1_% from 59.3 ± 7.5 to 68.1 ± 7.3, as comparing baseline to 12 months of treatment. CRP decreased only after 12 months of treatment (5.9 ± 2.3 mg/dL) as compared with the pre-treatment values (7.3 ± 1.6 mg/dL), although not significantly.

**Figure 2:**
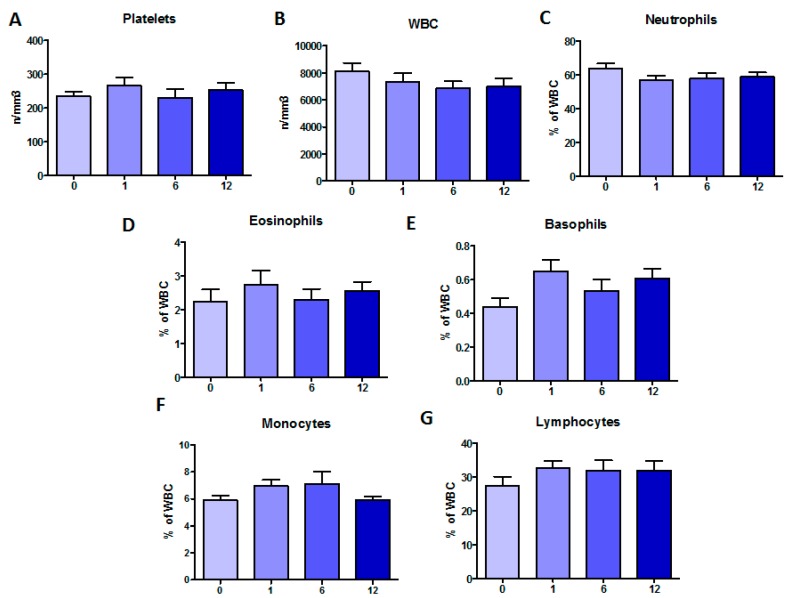
Changes in marrow-derived peripheral cells during Orkambi^®^ treatment: (**A**) platelets (n/mm^3^); (**B**) total WBC (n/mm^3^); (**C**) neutrophils (% of WBC); (**D**) eosinophils (% of WBC); (**E**) basophils (% of WBC); (**F**) monocytes (% of WBC); and (**G**) lymphocytes (% of WBC). Data are shown as mean ± SEM.

To evaluate a systemic effect of Orkambi^®^ on marrow-derived peripheral cells, the total number of platelets as well as white blood cells (WBC) and percentages of different cell populations were evaluated (Figure 2). Platelet counts did not show any substantial changes in time. WBC counts decreased at one month post-treatment and stabilized from six months onward. Neutrophils followed the same trend. Eosinophils and basophils increased at one month post-treatment, but while eosinophils returned to pre-treatment values, basophils were higher than baseline also at longer times. Finally, while monocytes peaked at six months post-treatment and then decreased to pre-treatment levels, lymphocytes increased at one month of treatment and remained at these levels up to 12 months. None of these changes were significant.

An extension study was conducted on clinical and laboratory parameters by enlarging the analyses to 20 other patients homozygous for *F508del* taking Orkambi^®^ as additional therapy and thus obtaining a total of 34 CF patients. All parameters confirmed the trend observed with only 14 patients, however none of these changes was significant (Table S1).

### 2.2. Effect of the Treatment with Orkambi^®^ on CFTR-Dependent Chloride Efflux and Protein in MNCs

In the cohort of 14 patients, we analyzed whether treatment with Orkambi^®^ could influence CFTR-dependent chloride efflux in circulating MNCs. CFTR-dependent chloride efflux was analyzed by spectrofluorimetric analysis using the chloride-sensitive dye, MQAE, following a procedure that we developed [22]. The experimental conditions for the CFTR functional assay was established with MNCs obtained from healthy subjects. Briefly, chloride efflux rate after substitution of chloride by nitrate in the perfusion medium was measured by the change in fluorescence (∆(F/F0)/min) of MQAE. Stimulation of PKA by addition of FSK plus IBMX increased chloride efflux in MNCs. The addition of the CFTR specific inhibitor, CFTRinh-172 (5 µM), before and during the next stimulus inhibited this increase to basal levels, confirming the specificity of the effect on CFTR-mediated anion transport. CFTR-dependent chloride efflux is defined as the difference between the rate of FSK plus IBMX-stimulated chloride efflux in the absence and presence of CFTRinh-172 treatment.

**Figure 3:**
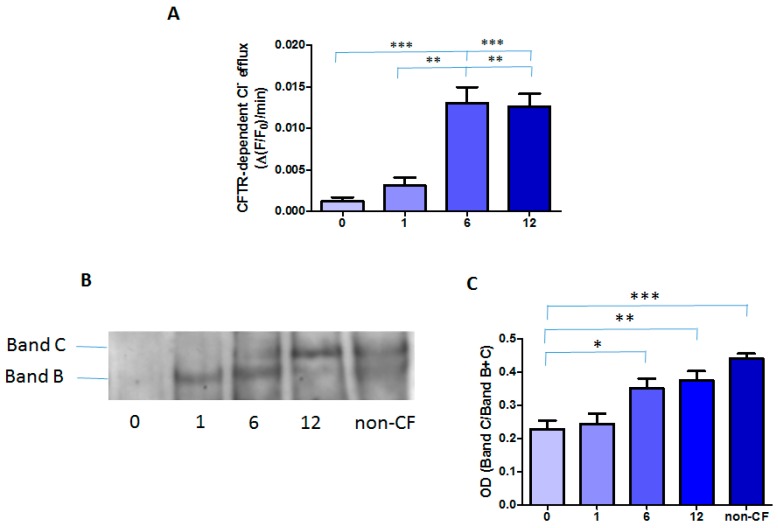
CFTR activity and protein in MNCs: (**A**) cumulative data of CFTR activity obtained from 14 patients; (**B)** a representative Western blot of CFTR protein; and (**C**) densitometric analysis of Western blots represented as the OD ratio of Band C over Bands B and C in three CF subjects and in a pool derived from five non-CF subjects. Data are shown as mean ± SEM. * *p* < 0.05, ** *p* < 0.01, *** *p* < 0.001.

Treatment with Orkambi^®^ increased CFTR-mediated chloride efflux in all patients, although they responded differently (Figure S1). As shown in Figure 3A, CFTR-dependent chloride efflux was not detectable in MNCs prior to treatment, in line with our previously published data [23]. Cumulative data show that the CFTR-dependent chloride efflux in MNCs was significantly increased at 6 (0.0130 ± 0.0019 ∆(F/F0)/min) and 12 months (0.0126 ± 0.0015 ∆(F/F0)/min) post-treatment, although still being lower than observed in non-CF MNCs (0.0187 ± 0.0022 ∆(F/F0)/min, *n* = 6).

We then determined the effect of Orkambi^®^ treatment on the expression/maturation of F508del- CFTR protein. As shown in Figure 3B, in the MNCs pool derived from healthy subjects (n = 5), wt CFTR was expressed as the fully glycosylated mature form of the protein (Band C) migrating at 180 kDa and the core glycosylated form, Band B (160 kDa). Importantly, while before Orkambi^®^ treatment the CF patient-derived MNCs expressed only a barely detectable immature band B of F508del-CFTR, after 6 and 12 months of treatment, there was an increase in the expression also of the mature band C of F508del CFTR protein (Figure 3B). Densitometric analysis revealed that Orkambi^®^ treatment resulted in a significant increase of Band C in CF MNCs at 6 and 12 months and that the CFTR protein amount at 12 months was similar to that of non-CF MNCs (Figure 3C).

### 2.3. Correlation among the Clinical Parameters and CFTR Activity

**Figure 4:**
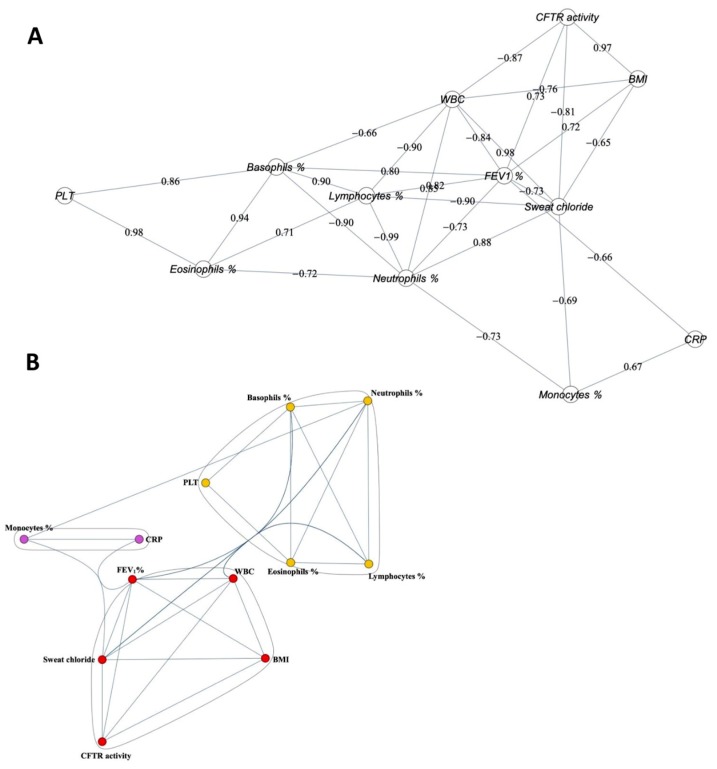
Correlations and cluster analysis over all time points during Orkambi treatment: (**A**) correlation graph; and (**B**) cluster analysis.

To determine whether the parameters were correlated with each other during Orkambi^®^ treatment, we performed a correlation analysis of the parameters’ behavior over treatment time, considering the average value of each parameter on all patients. A representation of interrelation of the variables with each other demonstrated that CFTR correlated with BMI, WBC, FEV_1_, and sweat chloride (Figure 4A). Only the correlation of CFTR activity with BMI was significant (r = 0.97; *p* < 0.05). WBC strongly correlated with sweat chloride (r = 0.98; *p* < 0.05). Figure 4A also shows that, while lymphocytes correlated positively with basophils and eosinophils, they strongly negatively correlated only with neutrophils (*r* = −0.99; *p* < 0.01). Platelets and eosinophils correlated positively (r = 0.98; *p* < 0.05). Monocytes and CRP correlated with low statistical significance.

From the above correlations, we can highlight three well-defined groups (Figure 4B): (i) CFTR activity, FEV_1_, sweat chloride, WBC, and BMI; (ii) platelets, neutrophils, lymphocytes, basophils, and eosinophils; and (iii) CRP and monocytes.

## 3. Discussion

The effects of CFTR modulators on pulmonary function, pulmonary exacerbations, sweat chloride concentration, and quality of life have been well documented, whereas less evidence is available on extrapulmonary manifestations, including inflammation and immunity [24]. A few studies have shown that ivacaftor increases innate immune cell activities, such as killing of *P. aeruginosa* by neutrophils and monocytes [25,26] and oxidative burst of neutrophils as mediated by enhanced expression of hydrogen voltage-gated channel-1 [22]. Interestingly, ivacaftor-lumacaftor (Orkambi^®^) treatment was not associated with improved phagocytosis and killing of *P. aeruginosa* by monocyte-derived macrophages [26]. Others have reported a decreased neutrophil–epithelial cell binding (which may prelude to decreased diapedesis) [27], while a proteomic analysis of monocytes from patients treated with ivacaftor showed a decrease in the expression of markers that mediate migration and response to interferon-γ [28].

As a novel biomarker for therapeutic efficacy in CF patients focused on immune cells, we investigated if and how CFTR-dependent chloride channel activity in MNCs was modified by ivacaftor in the past [22] and by Orkambi^®^ in the present study. Ivacaftor treatment of patients harboring non-*G551D* gating mutations was associated with a significant increase in MNC CFTR channel activity and this response was paralleled by a decrease in sweat chloride concentration [22]. In the present study, CFTR channel activity increased significantly after 6 and 12 months of treatment with Orkambi^®^ of *F508del*/*F508del* patients while sweat chloride showed a significant decrease only at 6 months. Although the cohort of patients in both studies is very small (seven patients with ivacaftor and fourteen with Orkambi^®^), together with previously reported studies, it is tempting to speculate that CFTR modulators have variable effects depending on class mutation or on the extent to which the CFTR modulator increases CFTR activity. Importantly, in both studies, the only significant variations were observed for in vivo and ex vivo parameters determined directly by CFTR activity (sweat chloride and chloride channel in MNCs). Recently, it has been shown that complex alleles comprising *F508del* and an exonic variant (*F87L-I1027T*) can affect the increase in FEV_1_ in patients treated with Orkambi^®^ and the rescue of CFTR activity by lumacaftor in in vitro experiments [21]. Unfortunately, the number of patients in our study with a complex allele (i.e., with the mutation *A238V* in cis) was only n = 2 and this hampered the possibility to draw any strong appraisal of its effect on CFTR activity in MNCs.

While in the previous study with ivacaftor treatment, chloride MNC positively correlated with FEV_1_ and FVC [22], in the present study, MNC CFTR activity correlated with BMI. Two phase 3 randomized clinical trials (TRAFFIC and TRANSPORT) investigated the comparative safety and efficacy of Orkambi^®^ in patients with CF who were 12 years old and older with mild to moderate lung disease and who were homozygous for the *F508del*-CFTR mutation [29]. Both studies demonstrated that 24 weeks of treatment with Orkambi^®^ was associated with statistically significant improvements in FEV_1_% predicted (absolute increases of 2.6–3.0% and relative increases of 4.3–4.5%); however, the magnitude of improvement in respiratory function is uncertain but may be clinically relevant for CF patients, given that changes in FEV_1_% have been shown to be correlated with mortality [30]. An ongoing extension study (PROGRESS) demonstrated that the improvements in FEV_1_% persisted after 48 weeks of treatment [18]. In this study, the absolute increase in FEV_1_% at 12 months (i.e., 48 weeks) was 8.9%, making this improvement higher than in previous clinical trials, although not significant as compared to pre-therapy values, likely because we dealt with 14 patients instead of hundreds in phase 3 studies. To see if an increase in patients’ number could yield a different result, we extended the analyses of clinical parameters to 20 other patients, however resulting in no statistically significant changes. Our cohort of 34 patients comprises subjects with a broad respiratory function at the baseline (from 21 to 118 FEV_1_%), and this may constitute a big difference with phase 3 studies, in which patients were initially excluded if their baseline lung function (FEV1%) was less than 40%, as also has been observed in a recent observational study [31]. Again, this consideration indicates conducting observational studies in a higher number of patients with more homogenous clinical status. Nevertheless, a real-world trial on severe CF patients has shown that about one third of all patients enrolled to receive Orkambi^®^ had an absolute change in FEV_1_% of 5% or more in comparison to baseline and 13% had an increase of at least 10% in FEV_1_% [32], indicating that Orkambi^®^ treatment may result in beneficial effects on lung function comparable to those reported in clinical trials in patients with less severe lung disease, as in our study. Results for change from baseline in BMI and weight gain were inconsistent with statistically significant improvements observed in TRANSPORT but not in TRAFFIC studies [29]. A pre-planned pooled analysis, however, suggests that treatment with Orkambi^®^ was associated with improvements in BMI, although the magnitude of improvement was of uncertain clinical significance [29]. Here, we picked up a positive correlation with BMI that was confirmed by the clustering analysis in which we found that CFTR activity in MNCs composed a sub-group that included BMI, FEV_1_%, sweat chloride, and WBC, suggesting that this ex vivo activity related to CFTR is a variable that explains some in vivo features related to the respiratory function, sweat gland function, body growth, and inflammation.

It has been shown that CFTR dysfunction is associated with a slowing of inflammation resolution and an altered bacterial killing [33,34,35,36]. Whether our results showing the recovery of CFTR function in circulating MNCs upon Orkambi^®^ treatment is reflected by a protective behavior of monocytes/macrophages in the CF lung will be the objective of future studies.

In conclusion, our real-life study suggests CFTR activity in circulating mononuclear cells as an interesting surrogate biomarker of the adjunctive therapy of CF patients with Orkambi^®^. In particular, young CF patients who typically have both mild disease and poorly standardized outcome measure may benefit from the development of more sensitive disease biomarkers.

## 4. Patients and Methods

### 4.1. Patients

All patients enrolled in this study were homozygous for the *F508del* mutation and had a confirmed diagnosis of CF [23]. The first cohort of 14 patients was comprised of people ≥12 years old (5 females, 9 males). These patients commenced Orkambi^®^ at the median age of 23.5 years (range 13–45 years). Two patients also had the *A238V* mutation in cis [24]. Twenty other patients (14 females, 6 males) were also treated with Orkambi^®^ and included in this study (median age of 16.5 years; range 6–32 years). Two out of 20 also had the *A238V* mutation in cis. At the first dose of Orkambi^®^, all patients were pancreatic insufficient. Overall, sixteen patients were colonized in the airways by *Staphylococcus aureus*, 13 by *Pseudomonas aeruginosa*, 9 by *Candida albicans*, 4 by *Stenotrophomonas maltophilia*, 2 by *Enterobacter cloachae*, 1 by *Klebsiella pneumoniae*, 1 by *Klebsiella oxytoca*, 1 by *Candida lusitanae*, 1 by *Achromobacter xylosoxidans*, 1 by *Nocardia*, and 1 by *Burkholderia cepacia*. Patients enrolled in this study took two capsules of Orkambi^®^ every 12 h (each capsule containing 200 mg of lumacaftor and 125 mg of ivacaftor) in association with 20 g of fat. All the other medications were not discontinued. At baseline (pre-therapy) and at 1, 6, and 12 months of treatment the following outcome measures were considered: sweat chloride (mEq/L), FEV_1_%, body mass index (BMI; kg/m^2^), C-reactive protein (CRP; mg/dL), platelets (n/mm^3^), white blood cells (WBC; n/mm^3^), neutrophils (% of WBC), basophils (% of WBC), eosinophils (% of WBC), lymphocytes (% of WBC), and monocytes (% of WBC). Moreover, CFTR-dependent chloride efflux in mononuclear cells (MNCs) was considered.

The study was approved by the ethics committee of the Azienda Ospedaliera Universitaria “Policlinico” of Bari, Italy (No. 999/CE/08/01/2016) and performed in accordance with the 1964 Declaration of Helsinki. Written informed consent from the adult study subjects or written consent from the next of kin, caretakers, or guardians on behalf of the enrolled children was obtained.

### 4.2. Isolation of Blood Mononuclear Cells

Heparin anti-coagulated blood was obtained from the 14 CF patients in accordance with the ethical guidelines. Briefly, peripheral blood mononuclear cells were isolated from dextran sedimentation followed by Ficoll-Hypaque (GE Healthcare, Little Chalfont, UK) density gradient centrifugation. The upper plasma layer was aspired and the middle phase containing mononuclear cells was collected and washed in RPMI 1640 medium (Aurogene, Roma, Italy). Viability of the cells was determined by Trypan Blue dye extrusion and resulted in ≥98% viable MNCs.

### 4.3. CFTR Function Measurement in Mononuclear Cells

First, 4 × 10^5^ freshly isolated mononuclear cells (MNC), seeded on 0.1% poly-l-lysine coated glass coverslips, were loaded overnight in culture medium containing 5 mM N-(ethoxycarbonylmethyl)-6-methoxyquinolinium bromide (MQAE) at 37 °C in a CO_2_ incubator. Fluorescence was recorded with a Cary Eclipse Varian spectrofluorometer. To measure chloride efflux rate across the plasma membrane, the perfusion medium was changed with a medium in which chloride was substituted with iso-osmotic nitrate as previously described by us [22]. All experiments were performed at 37 °C in HEPES-buffered bicarbonate-free media, Cl^−^ medium (in mM: 135 NaCl, 3 KCl, 1.8 CaCl_2_, 0.8 MgSO_4_, 20 HEPES, 1 KH_2_PO4, and 11 glucose) and Cl- free medium (in mM: 135 NaNO_3_, 3 KNO_3_, 0.8 MgSO_4_, 1 KH_2_PO_4_, 20 HEPES, 5 Ca(NO_3_)_2_, and 11 glucose). At the end of each experiment, a two-point calibration procedure was performed: the maximal intensity of fluorescence (F0) was determined by perfusing the cells with the Cl^−^ free medium; the minimal fluorescence was obtained by exposing the cells to a solution containing (in mM) 110 KSCN, 1 MgSO_4_, 10 HEPES, 1 CaSO_4_, 5 glucose, and 0.005 valinomycin. The rate of chloride efflux induced by treatment with forskolin plus IBMX (3-isobutyl-1-methylxanthine) after substitution of chloride by nitrate in the perfusion medium was measured by the change in fluorescence of MQAE. CFTR-dependent chloride efflux was calculated as the difference in alterations of FSK stimulated fluorescence in the absence and presence of CFTR selective inhibitor CFTRinh-172 (5 µM).

### 4.4. Western Blot of CFTR in MNCs

Protein extraction from mononuclear cells was performed essentially as described previously [23]. In total, 5 × 10^6^ MNCs were lysed in ice-cold buffer (pH 8.0) containing 50 mM NaCl, 50 mM Tris, 1 mM EDTA, 1% Triton X-100, 0.1 mM Na-Vanadate, 0.5 mM dithiothreitol, 0.5 mM phenylmethylsulfonyl fluoride, and 10 µL/mL of protease inhibitor cocktail (Sigma-Aldrich, Milan, Italy). Lysates were frozen overnight at −80 °C, and then cleared by centrifugation (22,000 *g*, 20 min, 4 °C).

Supernatants were collected and assayed for protein concentration using the Micro BCA Protein Assay kit (ThermoFisher Scientific, Monza, Italy). Equivalent amounts of protein (usually 20 µg) were supplemented with sample buffer (1.5 M Tris, pH 6.8) containing 56% sucrose, 14% SDS, 1.4% β-mercaptoethanol, and 0.02% bromophenol blue, and separated using a 4–12% polyacrylamide gel (Bio-Rad Laboratories, Milan, Italy). The gel was transferred to PVDF membranes Millipore (Merk Life Science, Milan, Italy) and processed for Western blotting by using monoclonal CFTR antibody (dilution 1:500; clone: 24–218 1, MAB25031 R&D Systems, Minneapolis, MN, USA) and anti-mouse HRP-conjugated secondary antibody. Immunocomplexes were detected with Pierce ECL plus reagent (ThermoFisher Scientific, Rodano (Milan), Italy) and image processing was carried out using Adobe Photoshop and the Image software package (version 1.61, National Institutes of Health, Bethesda, MD, USA). As the housekeeping proteins themselves were variably expressed, the total lane density of transferred proteins on the membrane stained with Ponceau red was used for the normalization of proteins. Due to limited amount of MNC, CFTR protein analysis was carried out only in three CF patients.

### 4.5. Statistical analysis

The initial dataset was composed of four-stage recordings (pre-therapy, 1 month, 6 months, and 12 months) for 14 patients and for each of the 12 parameters detected (WBC, neutrophils, lymphocytes, monocytes, basophils, eosinophils, platelets, CRP, FEV_1_%, BMI, sweat chloride, and CFTR activity). The missing data (less than 3% of the total) were imputed with the average of the values present for each parameter.

The initial detail values of the 14 patients were then summarized with the averages of the patient records for each of the 4 times and 12 parameters, for a total of 48 final values. A descriptive statistical analysis was performed on this dataset, which showed an almost always normal distribution of values for the parameters. To compute the p-values, analysis of variance with post-hoc Tukey test was used. Then, the Pearson’s correlation coefficient matrix among the 12 parameters was calculated (see the equivalent correlation graph shown in Figure 4A). After establishing a threshold of 0.65, all links (and their nodes) with a coefficient lower than the threshold were removed from the correlation graph. On the remaining subgraph, a clustering analysis was carried out (by means of the method of modularity maximization) identifying three communities of parameters (with a greater number of connections between the vertexes of the same community and relatively few that unite the vertexes of different communities), which are illustrated in Figure 4B.

## Figures and Tables

**Table 1:** Clinical characteristics of enrolled patients (*n* = 14) before Orkambi^®^ treatment.

	Min. Value	Median	Max. Value	25th Percentile	75th Percentile
Sweat chloride (mEq/L)	97.0	123.5	139.0	116.5	132.0
FEV_1_%	21.5	51.4	101.6	39.0	88.3
BMI (kg/m^2^)	16.7	20.6	23.9	18.5	21.8
CRP (mg/dL)	2.9	3.9	18.5	2.9	13.5

## Supplementary Materials

Supplementary Materials can be found at https://www.mdpi.com/1422-0067/21/7/2398/s1.

## Abbreviations

CFTR	Cystic fibrosis transmembrane conductance regulator
BMI	Body mass index
CRP	C-reactive protein
FEV_1_	Forced expiratory volume in 1 s
