# Supplementary material for: Treatment of Cystic Fibrosis Patients Homozygous for F508del with Lumacaftor-Ivacaftor (Orkambi®) Restores Defective CFTR Channel Function in Circulating Mononuclear Cells
Source: Int J Mol Sci. 2020 Mar 31;21(7):2398. doi: 10.3390/ijms21072398 (PMC7177453; doi:10.3390/ijms21072398)
Supplement: Supplementary file 1 [file ijms-21-02398-s001.zip › Supplementary material/Figure S1.pdf]

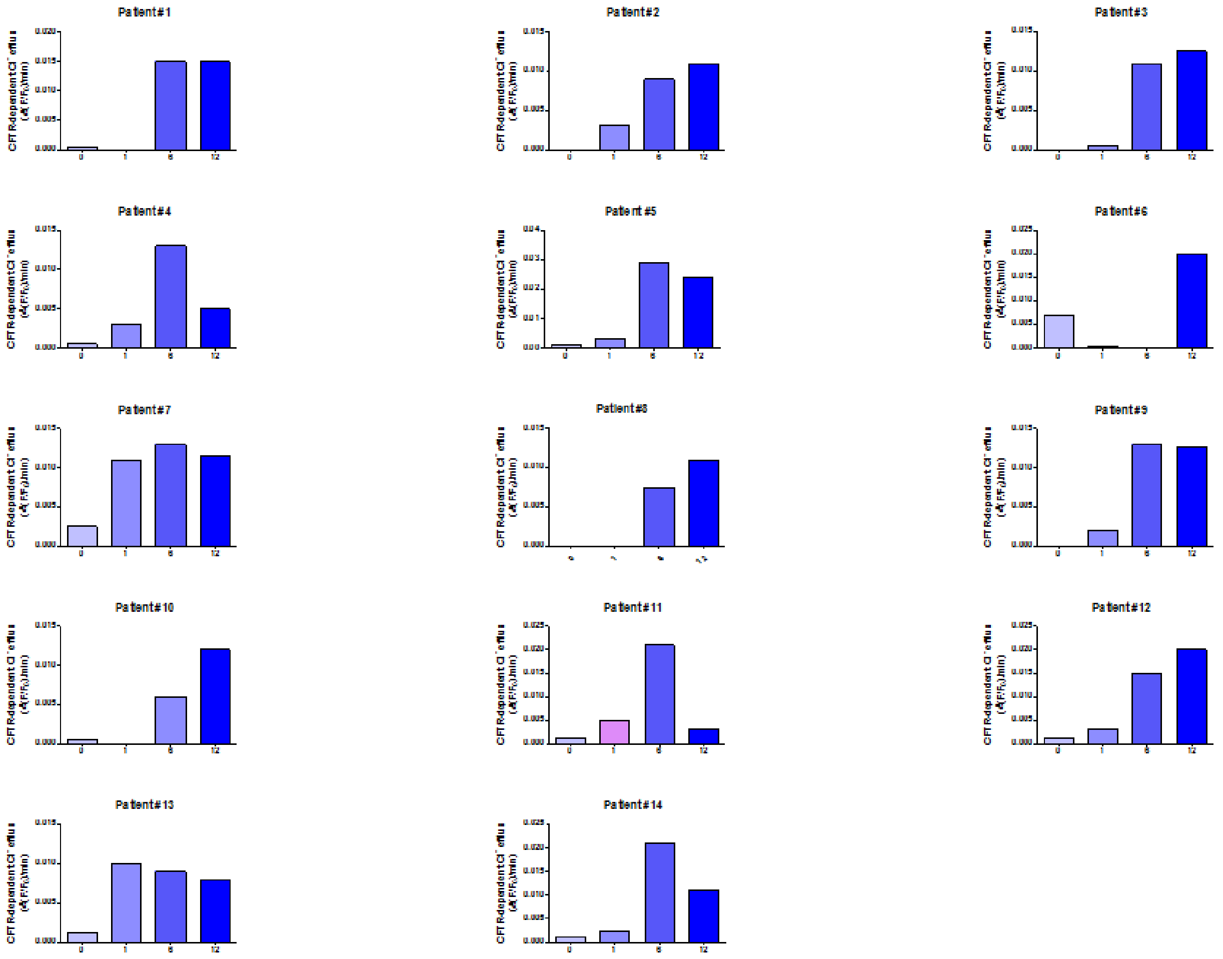

Figure S1. CFTR-dependent chloride efflux in circulating mononuclear cells. CFTR-dependent chloride efflux is shown in MNCs obtained from each CF patient at baseline (0), 1, 6, and 12 months from the beginning of Orkambi® treatment. While all patients are homozygous for the *F508del* mutation, patients #4 and patient #13 have also the *A238V* mutation in cis.
