# Supplementary material for: Treatment of Cystic Fibrosis Patients Homozygous for F508del with Lumacaftor-Ivacaftor (Orkambi®) Restores Defective CFTR Channel Function in Circulating Mononuclear Cells
Source: Int J Mol Sci. 2020 Mar 31;21(7):2398. doi: 10.3390/ijms21072398 (PMC7177453; doi:10.3390/ijms21072398)
Supplement: Supplementary file 1 [file ijms-21-02398-s001.zip › Supplementary material/Supplementary Table 1.docx]

Supplementary Table 1. Clinical parameters before and during Orkambi^®^ treatment in CF patients (n= 34)

|  | Pre-therapy | 1 month | 6 months | 12 months |
| --- | --- | --- | --- | --- |
|  |  |  |  |  |
| Sweat chloride (mEq/L) | 119.7 (2.8) | 105.5 (3.7) | 99.9 (2.8)*** | 106.7 (3.7) |
| FEV_1_ (% predicted) | 67.5 (4.7) | 75.3 (5.0) | 76.3 (5.2) | 75.9 (4.9) |
| BMI (kg/m^2^) | 19.6 (0.5) | 19.6 (0.4) | 20.5 (0.5) | 20.7 (0.5) |
| CRP (mg/dL) | 6.0 (0.9) | 6.9 (1.3) | 6.8 (1.0) | 4.8 (1.0) |
| WBC (n/mm^3^) | 8155 (316) | 7994 (524) | 7053 (282) | 7208 (309) |
| Neutrophils (% of WBC) | 59.0 (2.0) | 55.7 (1.9) | 54.5 (2.2) | 56.0 (1.5) |
| Lymphocytes (% of WBC) | 32.1 (1.9) | 34.6 (1.8) | 35.0 (1.8) | 34.9 (1.5) |
| Monocytes (% of WBC) | 6.1 (0.3) | 6.4 (0.3) | 6.7 (0.5) | 6.1 (0.2) |
| Basophils (% of WBC) | 0.5 (0.03) | 0.6 (0.04) | 0.5 (0.04) | 0.6 (0.04) |
| Eosinophils (% of WBC) | 2.3 (0.2) | 2.6 (0.2) | 2.4 (0.2) | 2.5 (0.2) |
| Platelets (n/mm^3^) | 286.3 (14.3) | 311.7 (18.5) | 300.0 (17.4) | 301.0 (16.4) |

Data are shown as mean (SE). ***p<0.001 vs. pre-therapy values.
